# Supplementary material for: Diverse Marinimicrobia bacteria may mediate coupled biogeochemical cycles along eco-thermodynamic gradients
Source: Nat Commun. 2017 Nov 15;8:1507. doi: 10.1038/s41467-017-01376-9 (PMC5688066; doi:10.1038/s41467-017-01376-9)
Supplement: Supplementary file 1 — Supplementary Information [file 41467_2017_1376_MOESM1_ESM.pdf]

Supplementary Discussion

SAG origins and sequence properties

Samples for single-cell genome sequencing were collected from marine environments including the Gulf of Maine (1 m depth), HOT station ALOHA (25 m), South Atlantic Gyre (800 m), Northeastern Sub-Arctic Pacific (NESAP) (2000 m, dysoxic conditions) and Saanich Inlet (SI) (100 m, dysoxic; 150 m, suboxic; 185 m, sulfidic) as well as a methanogenic terephthalate bioreactor and Etoliko Lagoon sediments. Percent GC content of the 25 sequenced Marinimicrobia SAGs ranged from 30.0% to 47.8% across different clades, averaging 32.7% in oxic ocean environments, 38.9% in oxygen minimum zone (OMZ) and anoxic basin environments, and 46.5% in the bioreactor and lagoon sediment environments (Supplementary Data 1).

Phylogeny of single-cell amplified genomes

To place Marinimicrobia SAGs in phylogenetic context with previously identified clades, SAG-associated small subunit ribosomal (SSU rRNA) gene sequences were appended to a reference database of full-length SSU rRNA gene sequences and a maximum likelihood phylogenetic tree was constructed (Fig. 1). Small subunit rRNA gene sequences from marine SAGs were affiliated with 6 out of 13 previously defined Marinimicrobia clades. Four out of 8 SSU rRNA gene sequences from SI SAGs were affiliated with the SHBH114 clade1, while remaining SAGs from SI and the NESAP OMZ were affiliated with the Arctic96B-7-A and –B clades. Sequences from the South Atlantic Gyre SAGs were affiliated with HF770D10 and ZA3648c clades, and all 5 SSU rRNA gene sequences derived from surface ocean SAGs from Gulf of Maine and HOT were affiliated with the ZA3312c clade. Finally, sequences derived from the methanogenic terephthalate bioreactor reactor and Etoliko Lagoon SAGs formed a new clade, HMTAb91, branching between previously detected Marinimicrobia clades and more distant branches affiliated with the phyla *Caldithrix* and *Deferribacteres*. Additional publically available SSU rRNA gene sequences derived from other methanogenic environments (including sub-seafloor methane hydrates, tar oil contaminated aquifer sediments, and mesophilic sludge) were also found to branch with HMTAb91 indicating a more widespread distribution of this clade in reducing environments.

While SSU rRNA trees can be robust predictors of phylogenetic relationships among microorganisms, concatenated alignments of multiple common single copy marker genes often provide greater resolution for assessing the validity of these relationships across genomes. The PhyloPhlAn pipeline[5] places predicted protein sequences derived from genomic data within a phylogeny of 3,737 sequenced genomes using concatenated protein alignments of over 400 marker proteins. We used PhyloPhlAn to phylogenetically place the 25 Marinimicrobia SAGs within the bacterial domain (Supplementary Fig. 2) as well as to construct a de-novo SAG phylogeny with closely related reference organisms identified through SSU rRNA sequence clustering (Fig. 2A). PhyloPhlAn placed Marinimicrobia SAGs within the same clades identified using SSU rRNA-based clustering methods and confirmed that the phylum *Caldithrix abyssi* contains the closest related genomes to Marinimicrobia relative to Ignavibacteriae, Bacteroidetes, and Deinococcus-Thermus (Supplementary Fig. 2).

Genomic streamlining and core metabolic potential

In depth examination of individual SAGs and population genome bins indicated that all Marinimicrobia clades harbour genes encoding outer-membrane proteins and a complete (ZA3312c) or partial pathway for synthesizing lipopolysaccharide building block lipid A, suggesting that Marinimicrobia is a Gram-negative phylum (Supplementary Data 6). Genome size estimation from SAGs with >40% genome completeness based on essential single-copy genes revealed that most Marinimicrobia clades have relatively small estimated genome sizes (0.70-1.63Mb), with outliers indicated in Arctic96B-7 (2.08-2.44Mb) and SHBH1141 (2.94-4.29Mb). All clades presented high coding base percentage (89.99-97.13%) and low clusters of orthologous groups (COG) redundancy (1.08-1.16) (Supplementary Fig. 1B) in comparison to *Escherichia coli* str. K-12 (86.59% and 1.68) (Supplementary Fig. 1A). ZA3312c clade manifested particualry high genome reduction (0.70-1.47Mb genome size, 96.34-97.13% coding bases, and 1.08-1.25 average COG redundancy) more extreme than that of "*Ca.* Pelagibacter" (1.12-1.44Mb, 95.92-96.87%, and 1.20-1.31). This observation is consistent with other planktonic marine microbial taxa such as the SAR11 clade. While other Marinimicrobia lineages presented reduced genome sizes, evidence of genome streamlining was not as strong due to inconsistencies in the COG functions recovered and overall genome completeness (Supplementary Fig. 1B). Additional Marinimicrobia SAGs with greater completion my help

elucidate the extent of genomic streamlining in other clades. Interestingly, Marinimicrobia central metabolic pathways vary widely among clades. For sugar metabolism, we identified clades encoding the Embden-Meyerhof-Parnar (EMP) pathway (Arctic96B-7 and HMTAb91), non-oxidative pentose phosphate pathway (PPP) (ZA3312c), both EMP and PPP (SHBH1141), and complementary anaplerotic reactions for gluconeogenesis (all clades) (Supplementary Data 6). Only Arctic96B-7 and ZA3312c possessed a pyruvate kinase, which connects sugar metabolism to the tricarboxylic acid (TCA) cycle for respiratory sugar catabolism. All groups encoded a complete TCA cycle, except for ZA3648c and HMTAb91. On the other hand, we only observed capacity for autotrophic carbon fixation in SHBH1141 (specifically, citrate-lyase-mediated reductive TCA cycle) and motility in HMTAb91 (flagellum biosynthesis). Thus, respiratory sugar catabolism, carbon fixation, and motility are unique to specific Marinimicrobia clades, indicating diverse lifestyles within the phylum.

Denitrification genes in Marinimicrobia ZA3312c and SHBH1141

Marinimicrobia SAGs and population genome bins affiliated with *nosZ* encoding clades ZA3312c and SHBH1141 were carefully evaluated for the complete repertoire of denitrification genes based on IMG/M and MetaPathways annotations (results are present in Supplementary Data 6 and in the text). Within the SHBH1141 population genome bin there were five genes annotated as 'nitrate reductase' by MetaPathways, however blast results indicated only ~70% sequence similarity to nitrate reductase, thiosulfate reductase and other DMSO oxidoreductases, making this annotation ambiguous. Further analysis of the SHBH1141 and ZA3321c SAGs and population genome bins revealed no evidence of genes encoding accessory proteins for nitrate reductase (such as *narHI* or *J)* or periplasmic nitrate reductase (*napAB)* or other denitrification genes. Within, both SHBH1141 and ZA3312c genes encoding *nos* accessory proteins were identified including *nosL, nosD.* ZA3312c also contained *nosY*. Taken together, these observations are consistent with the potential to express a functional nitrous oxide reductase. Further corroborating evidence of several diverse microbial lineages harboring only the *nosZ* gene (and associated accessory proteins) and no other denitrification pathway genes is found in Sanford et al. 2012 where the authors catalogue many a-typical NosZ sequences found in diverse lineages, a subset of which have been functionally validated[1]. BLAST searches of the ZA3312c and SHBH1141 NosZ protein sequences against RefSeq show similarity to a-typical NosZ from

Aquimarina megaterium within the Flavobacteriaceae and *Melioribacter roseus* within the Ignavibacteriales respectively.

Marinimicrobia nosZ *expression*

To investigate the potential role of Marinimicrobia ZA3312c and SHBH1141 clades as biological sinks for N_2O we evaluated the distribution of Marinimicrobia *nosZ* in metagenomes and metatranscriptomes globally using FAST with >80% nucleotide identity cutoff (Supplementary Fig. 7). The ZA3312c clade *nosZ* was identified in TARA oceans metagenomic dataset in both oxic and anoxic samples, while in OMZs it was detected in oxic, dysoxic, suboxic and sulfidic samples. Expression of the Marinimicrobia ZA3312c *nosZ* gene was observed along the redoxcline in Saanich Inlet, while in OMZs it was predominantly detected in suboxic waters. The ubiquitous and abundant ammonia oxidizing *Thaumarchaeota* has been proposed as an important source of N_2O in the ocean, capable of producing increased N_2O under decreasing O_2 conditions. The detection of ZA3312c *nosZ* transcripts predominantly in suboxic waters (in OMZs other than Saanich Inlet) is consistent with metabolic coupling between *Thaumarchaeota* ZA3312c under O_2 limiting conditions. SHBH1141 clade *nosZ* appears only in OMZs and predominantly under sulfidic conditions, though in Saanich Inlet it exists throughout the water column. Expression of Marinimicrobia SHBH1141 *nosZ* was observed throughout the Saanich Inlet water column and in sulfidic and suboxic waters in Peru and ETSP OMZs respectively. Under suboxic or sulfidic conditions in OMZs the dominant source of N_2O is likely incomplete denitrification driven by the abundant SUP05 bacterial group indicating potential metabolic coupling between SUP05 and SHBH1141 to complete the nitrogen loss process.

Further exploration of metagenomes and metatranscriptomes over the Saanich Inlet time series (Supplementary Fig. 7) indicates a strong dichotomy between ZA3312c and SHBH1141 clades. Regression analysis using a negative binomial distribution indicates significant positive correlation (p-value 0.011) between ZA3312c and NO_3^- (regressor weight +0.081), and a significant negative correlation (p-value = 0.043) between ZA3312c and H_2S (regressor weight -0.317). With SHBH1141 there was a significant (p-value = 0.034) positive correlation between SHBH1141 and H_2S (regressor weight = +0.101) and less significant (p-value 0.082) negative correlation between SHBH1141 and O_2 (regressor weight -0.043). Interestingly, the metatranscriptome did not show this strong dichotomy. The ZA3312c *nosZ* exhibited somewhat

sporadic expression; likely due to lower sequencing coverage of the metatranscriptome compared to the metagenome (overall the RPKM values for ZA3312c *nosZ* were low; maximum 2.5 RPKM). Expression of ZA3312c *nosZ* corresponded primarily with renewal events and the influx of oxygenated nutrient-rich waters from outside the inlet, with the potential to transport active ZA3312c populations. The SHBH1141 *nosZ* showed more consistent expression throughout the water column. However, the read-mapping based approach shown in Fig. 4 and the assembled sequence alignment approach in Supplementary Fig. 7 in February 2011 are not completely in sync with one another. Read mapping shows very little expression from SHBH1141 but sequence alignment via FAST shows low levels of expression of SHBH1141 *nosZ* throughout the water column. This discrepancy likely arises from the differential specificities of the two approaches. Read mapping is highly specific and would detect only highly similar sequences while FAST-based sequence alignment (80% identity cut-off) is more permissive, detecting similar but not identical sequences. It is possible that the renewal event in Saanich Inlet in August 2010 instigated the proliferation of a different population of SHBH1141 with a divergent *nosZ* sequence. An additional point of interest is the detection of SHBH1141 *nosZ* (and in fact other SHBH1141 genes) in 10 m surface waters in Saanich Inlet, though expression is not observed. One possible explanation is that SHBH1141 could be particle associated, persisting in anaerobic micro-niches.

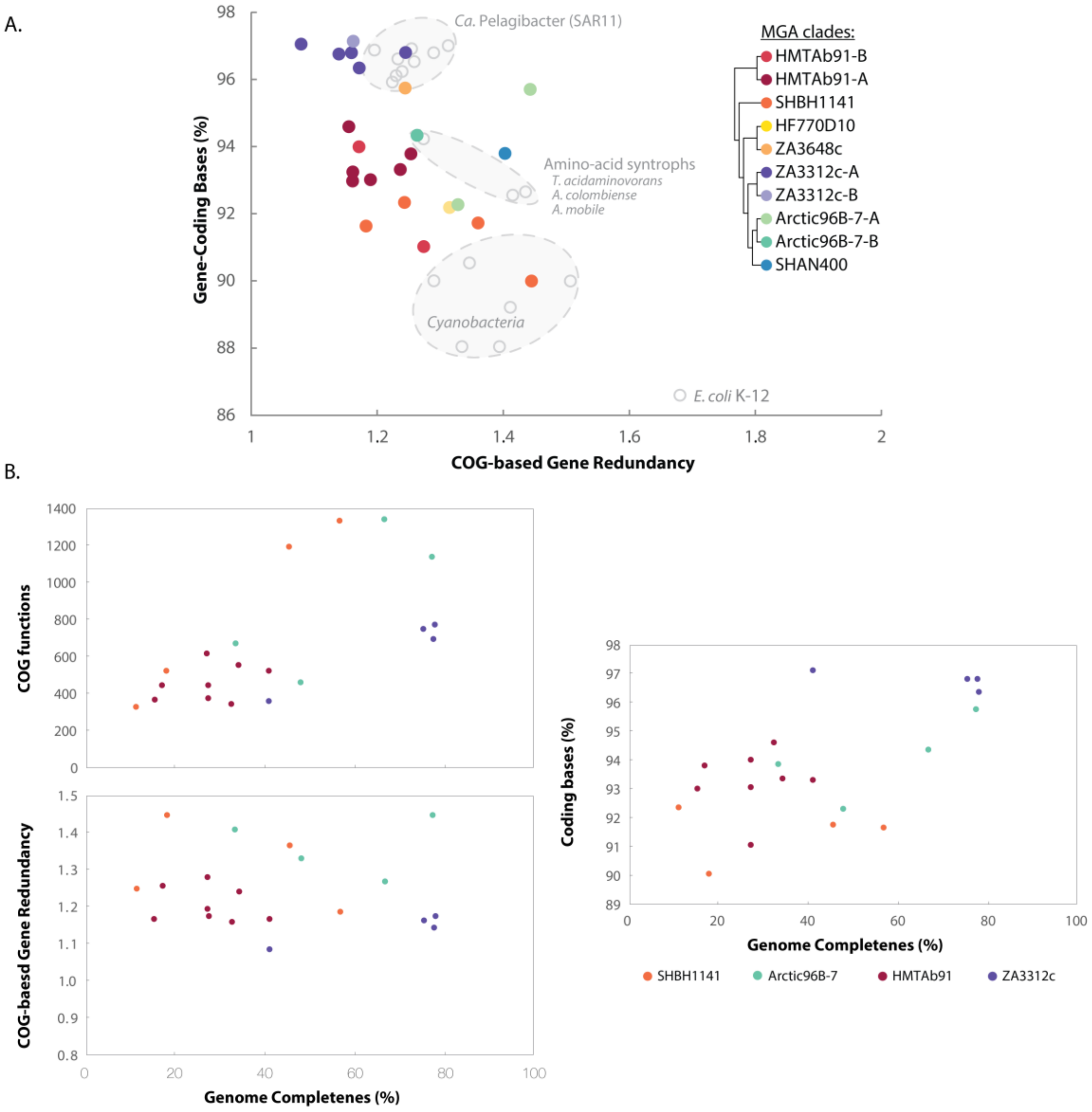

Supplementary Figure 1. Genomic streamlining in Marinimicrobia clades. (A) Comparison of genome reduction between Marinimicrobia clades and selected reference orgamisms based on estimated gene redundancy using clusters of orthologous groups (COG) annotation and frequency of gene-coding bases. **(B)** Benchmarking of genome reduction showing COG functions recovered, COG-based gene redundancy and percentage of coding bases as a function of estimated genome completeness.

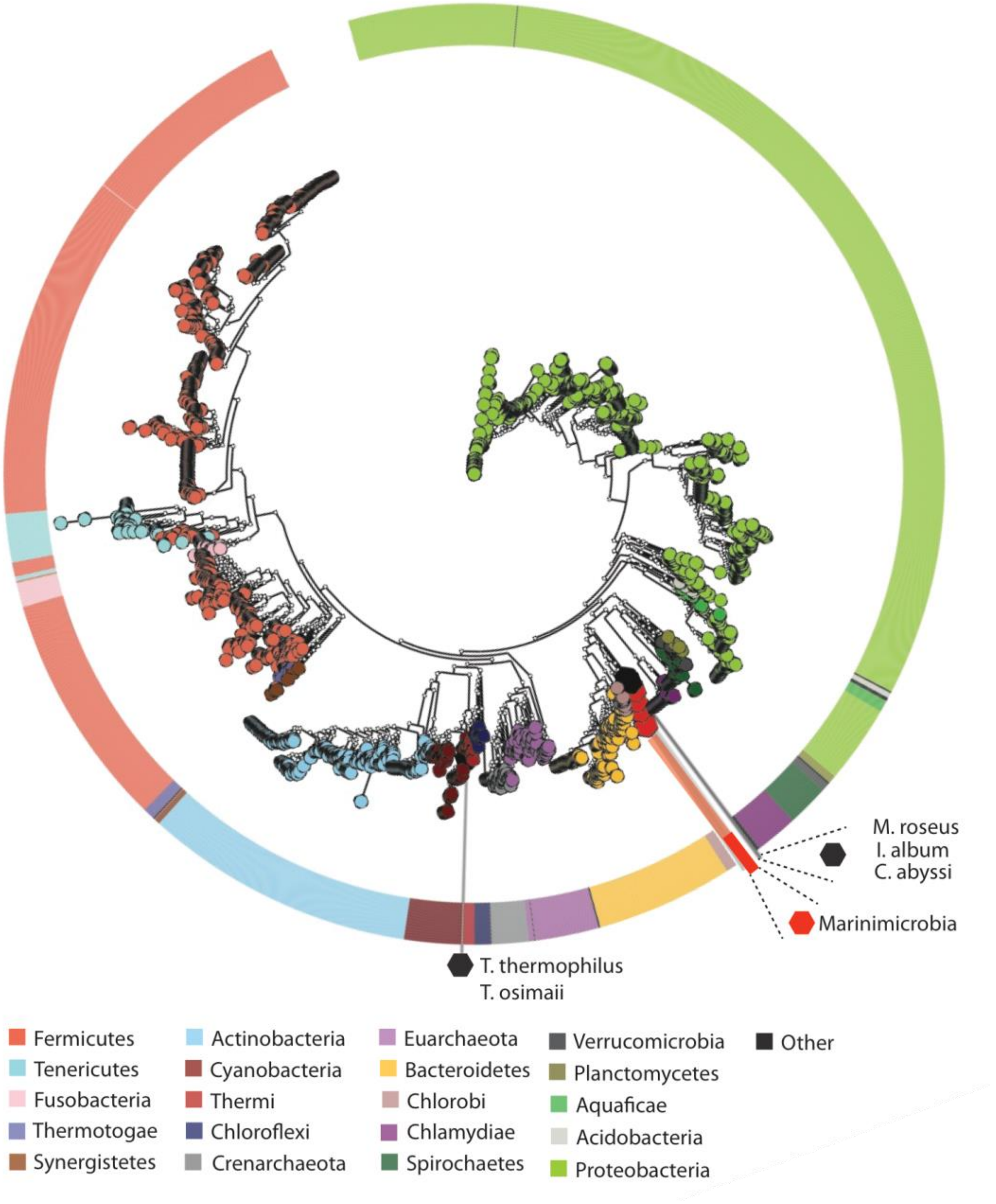

Supplementary Figure 2. **Marinimicrobia phylogenomic analysis.** Phylogenetic relationship of Marinimicrobia SAGs and related genomes within the microbial Tree of Life as determined by sequence alignment of 400 conserved protein sequences. The tree was generated using the PhyloPhlAn pipeline, placing Marinimicrobia SAG sequences within a phylogeny of 3,737 curated microbial genomes (colored by phylum). The 25 Marinimicrobia SAGs are shown as red hexagons, and 5 genomes previously identified as being highly similar to Marinimicrobia based on small subunit rRNA gene sequence shown as black hexagons.

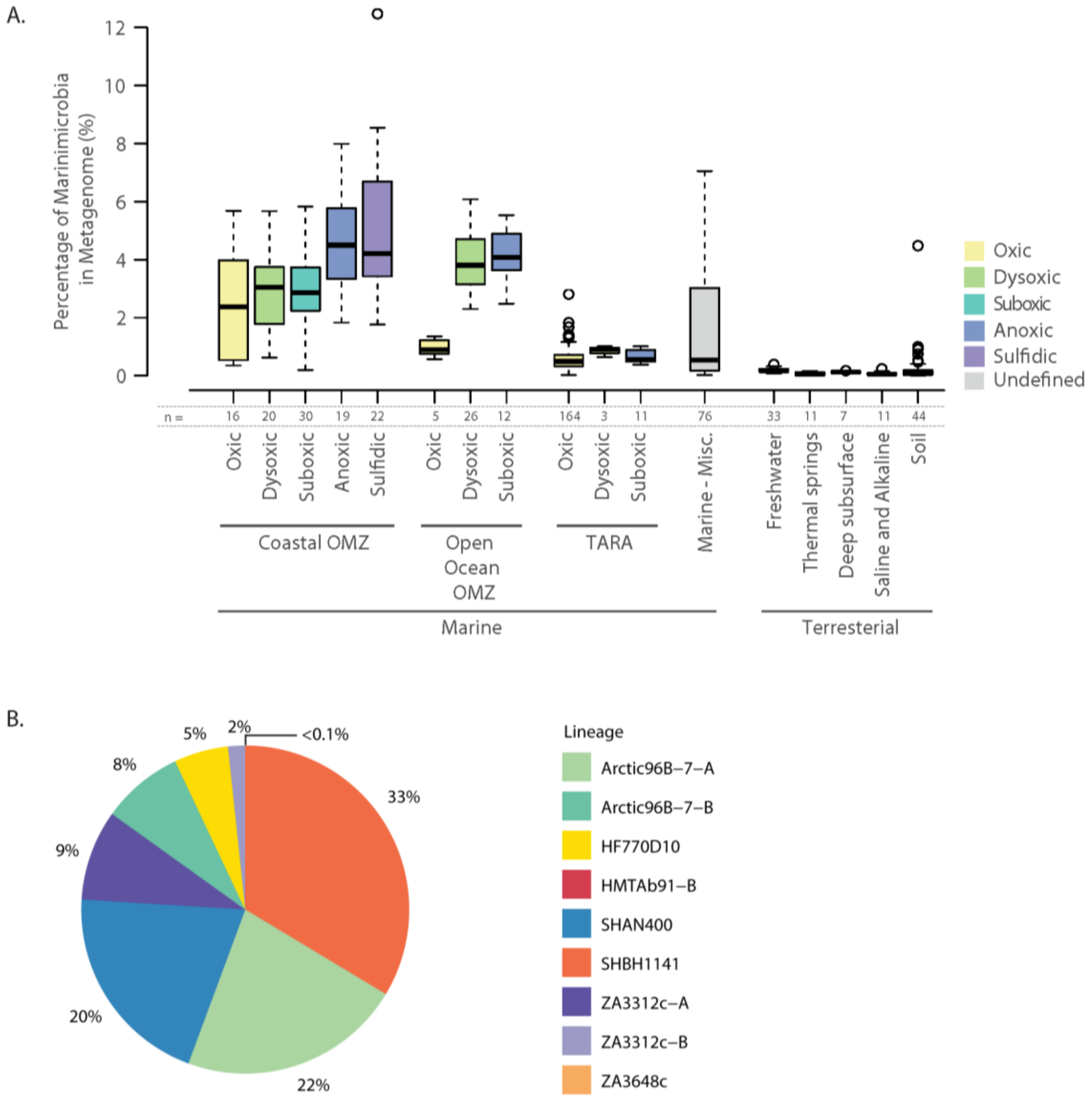

Supplementary Figure 3. Global prevalence of Marinimicrobia in public metagenomes. (A) Box and whisker plot showing the distribution of percentage of Marinimicrobia in surveyed metagenomes by region and redox status including Coastal OMZs (Saanich Inlet, Eastern Tropical South Pacific and the Peruvian upwelling system), Open Ocean OMZ (Northeastern subarctic Pacific), TARA oceans survey, miscellaneous marine and Terrestrial environments (see table S2) with the number of samples with Marinimicrobia present indicated as 'n ='. Where environmental data was available the metagenenomic sample was categorized as oxic (>90 μmol O2; yellow), dysoxic (20-90 μmol O2; teal), suboxic (1-20 μmol O2; blue), anoxic (<1 μmol O2), sulfidic (purple). (B) Global distribution of major Marinimicrobia clades.

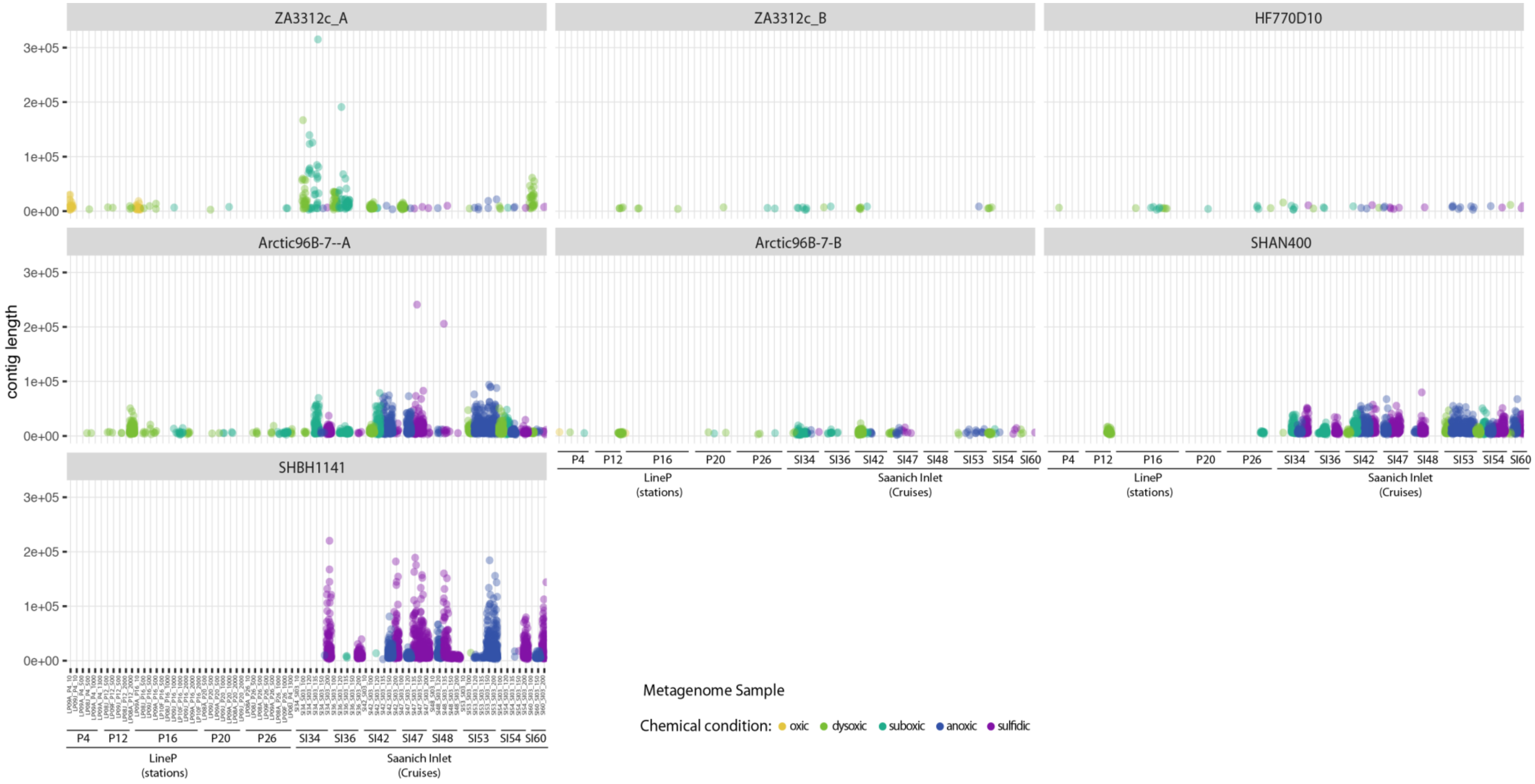

Supplementary Figure 4. Origin, length and abundance of contigs in population genome bins. Distribution of metagenomic contigs from Northeastern subarctic Pacific (NESAP) Ocean and Saanich Inlet (SI) metagenomic samples making up indicated SAG population genomes. Each dot represents a contig, sample origin is shown on the X-axis as indicated for NESAP and SI and contig length of recruited contigs is shown on the Y-axis. Colors represent the redox condition of the sample: oxic (>90 µmol O_2; yellow), dysoxic (20-90 µmol O_2; teal), suboxic (1-20 µmol O_2; blue), anoxic (<2 µmol O_2), sulfidic (purple).

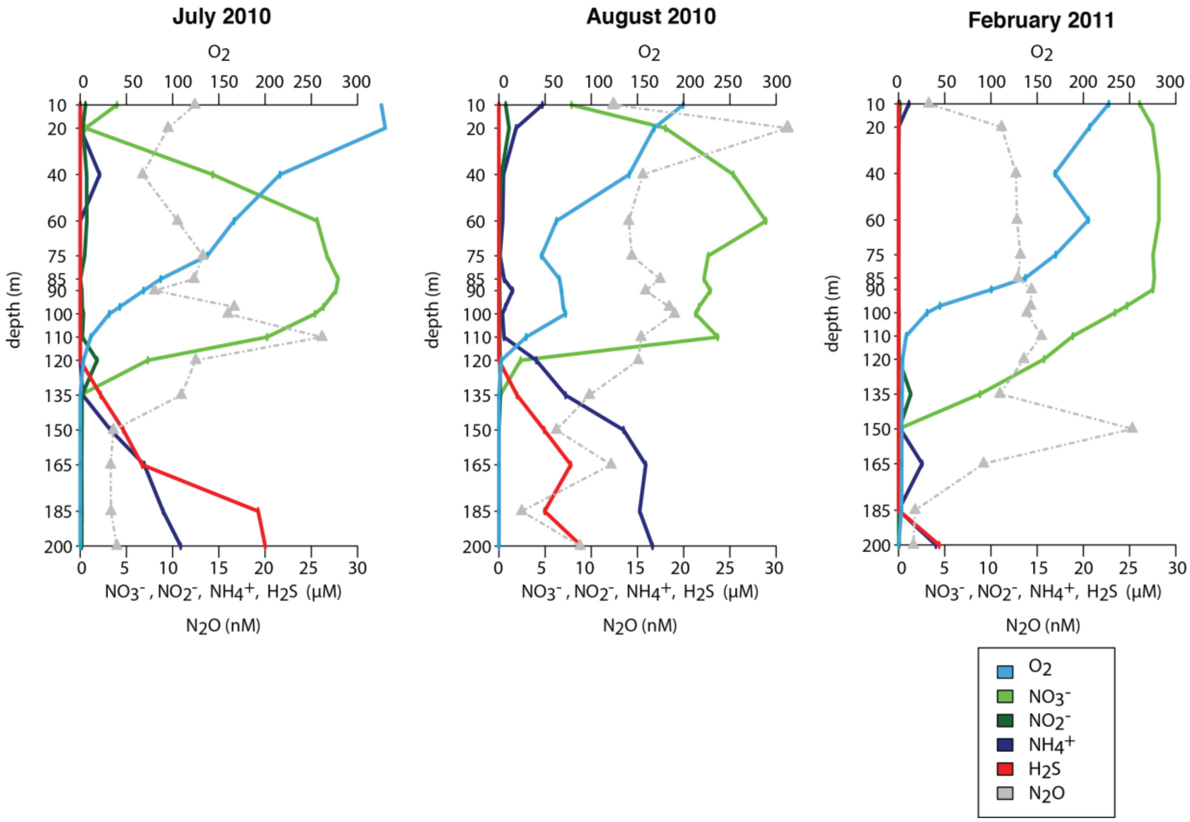

Supplementary Figure 5. Saanich Inlet water column chemistry associated with gene expression profiles. Plots of Saanich Inlet water column chemistry for time points used for metatranscriptomic expression analysis in Fig. 4.

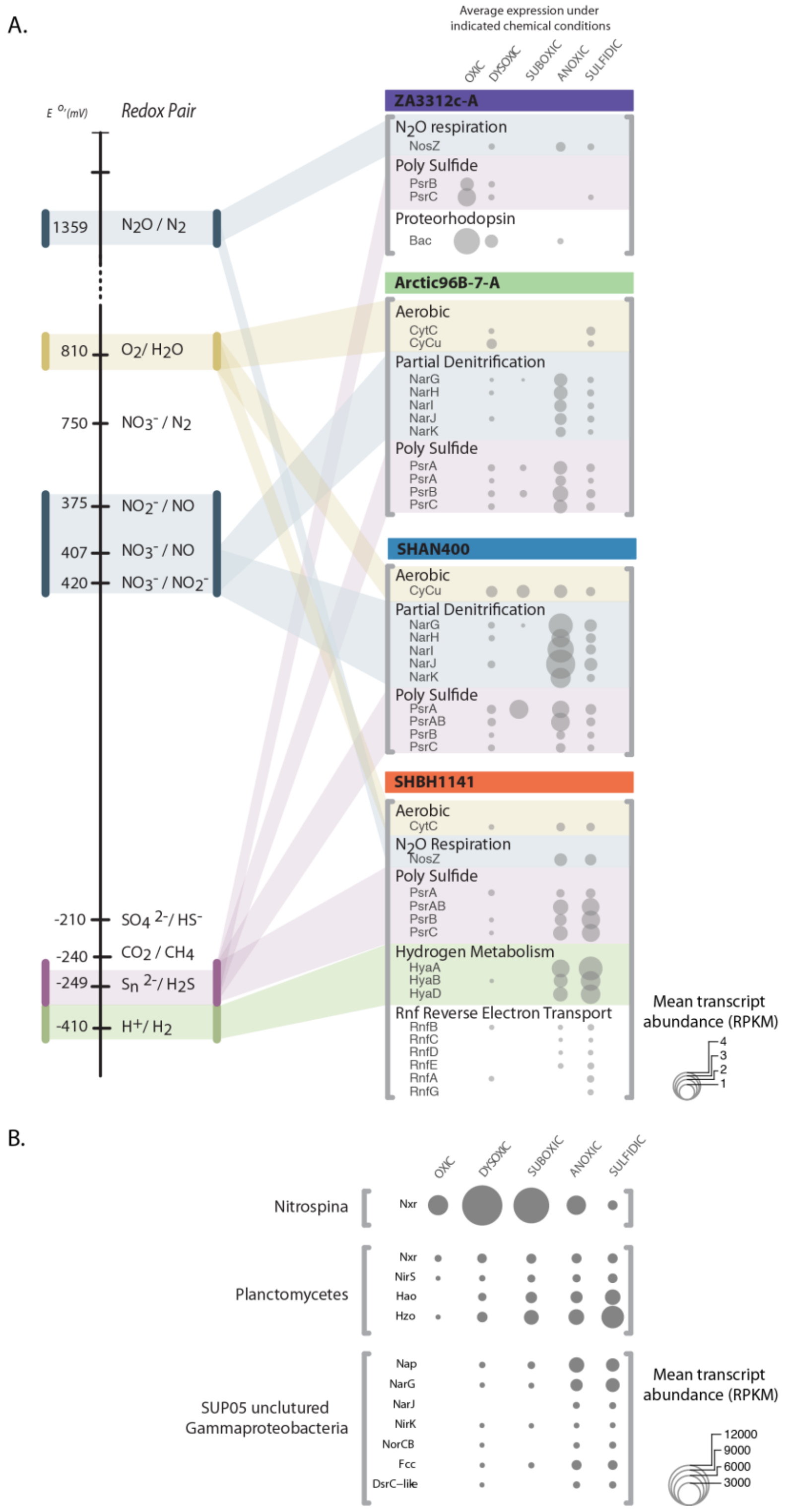

Supplementary Figure 6. Coupled patterns of gene expression. (**A**) Expression of Marinimicrobia energy metabolism genes across water column compartments (oxic, dysoxic, suboxic, anoxic, sulfidic) for July and August 2010 and February 2011 Saanich Inlet samples, mapped to redox pairs on the electron tower. (**B**) Expression of selected energy metabolism genes for potential co-metabolic partners.

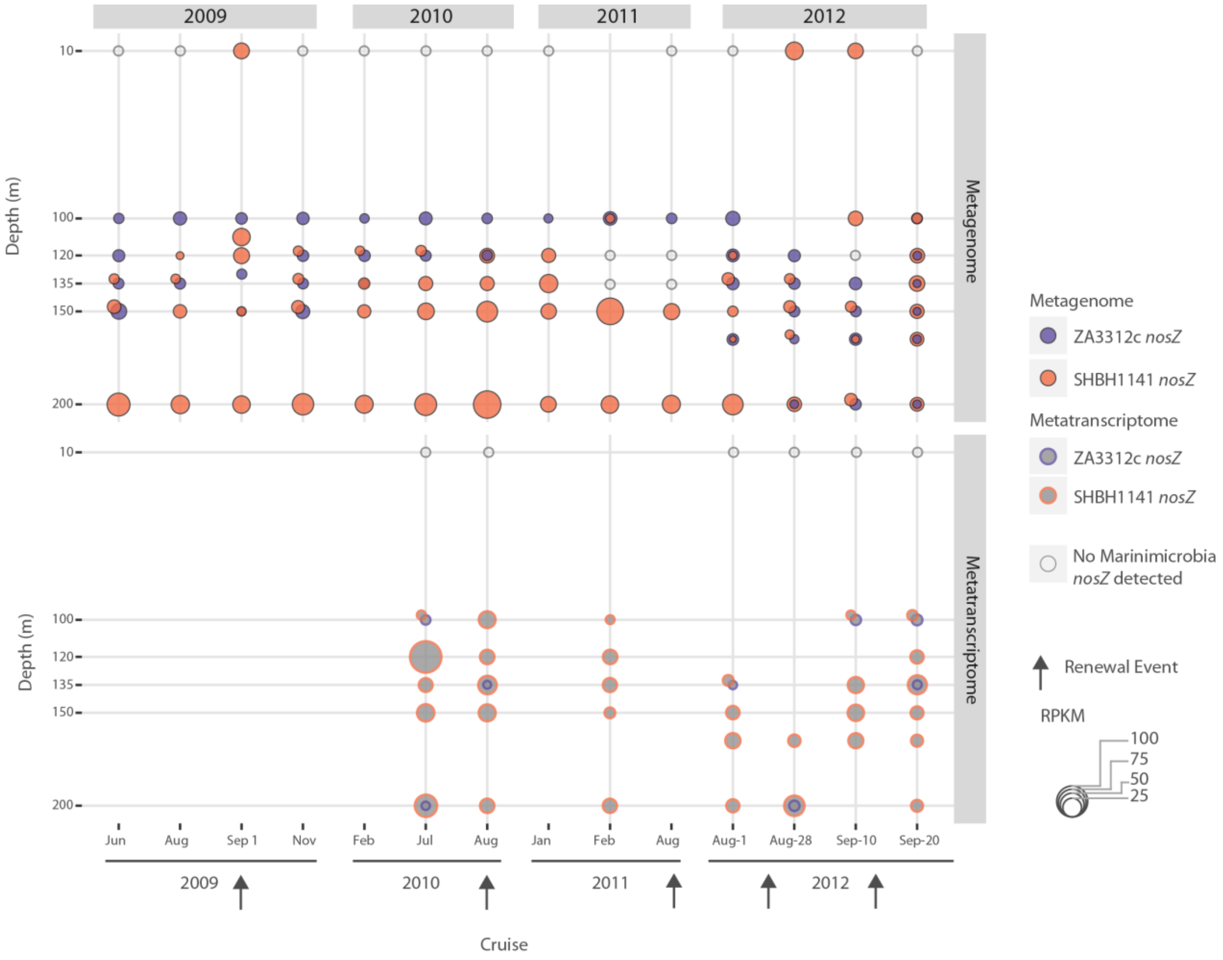

Supplementary Figure 7. Marinimicrobia *nosZ* abundance and expression in Saanich Inlet. Marinimicrobia *nosZ* abundance in Saanich Inlet time series metagenomes and metatranscriptomes. Dot size represents summed RPKM for each *nosZ* type in a given metagenome or metatranscriptome.

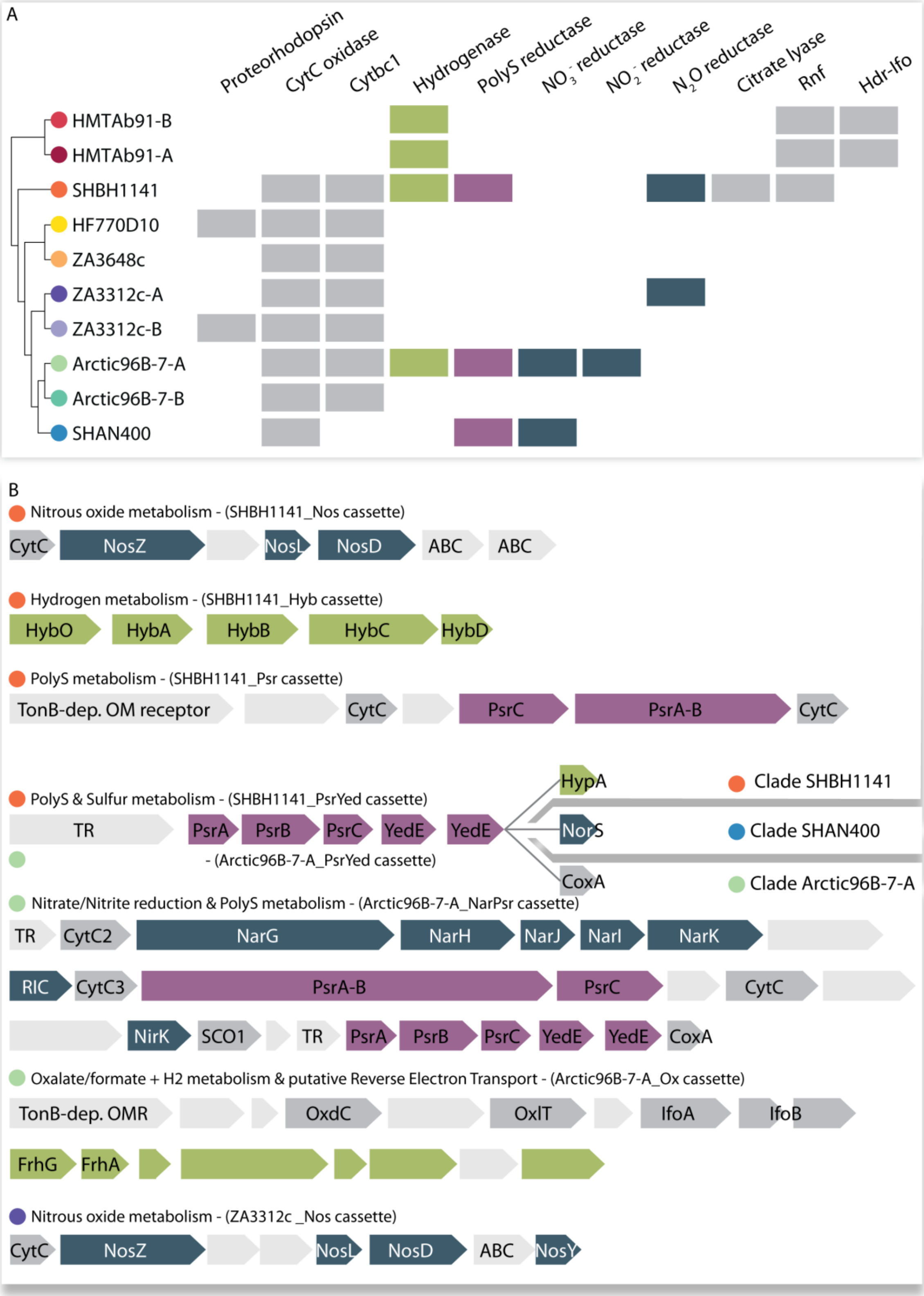

Supplementary Figure 8. Metabolic summary by operon organization. (**A**) Summary of energy metabolism, carbon fixation and co-metabolic interdependency (Rnf and Hdr-Ifo) for Marinimicrobia lineages. (**B**) Operons in Marinimicrobia SAGs showing different gene arrangements between clades.

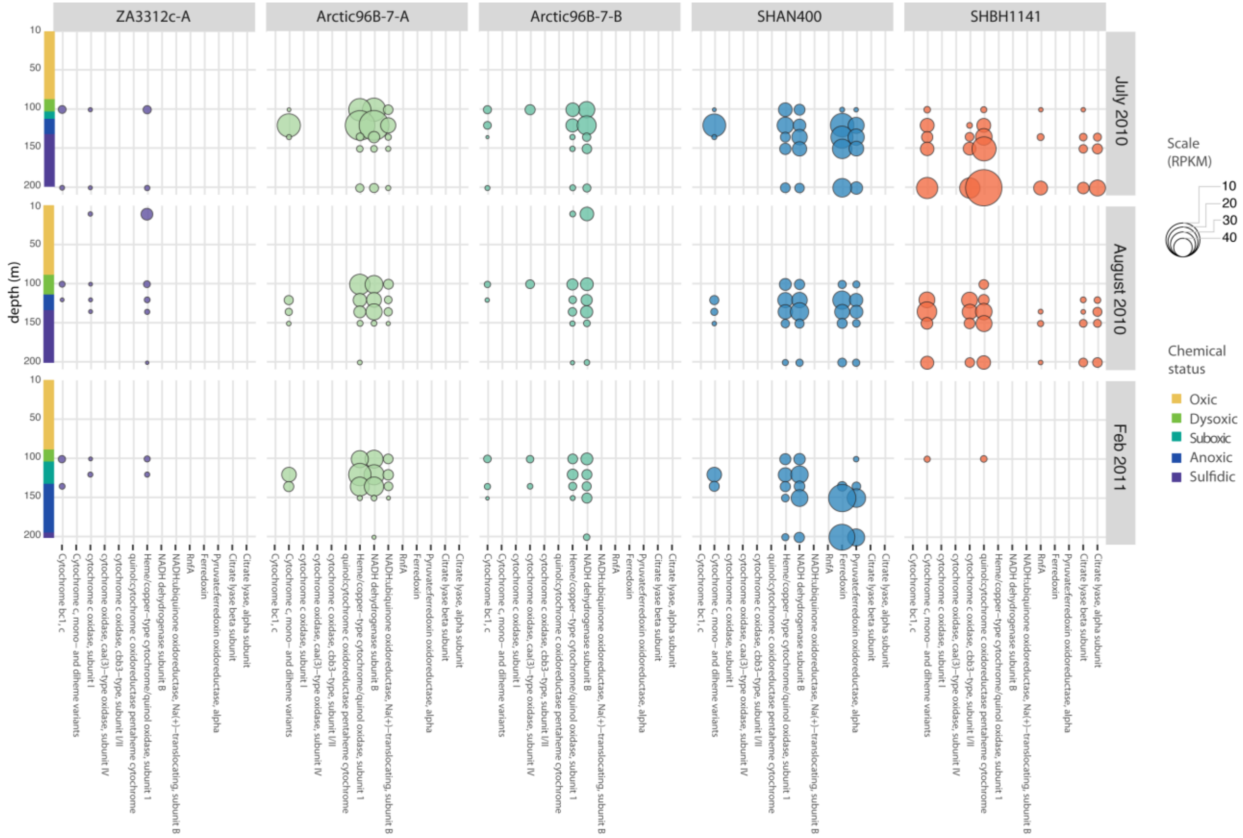

Supplementary Figure 9. Differential expression of enzymes involved in electron transfer in population genomes. Expression of selected enzymes involved in aerobic and anaerobic electron shuttling and energy production in Saanich Inlet station S3 at three time points from 10 to 200 m as reads per kilobase per million mapped (RPKM) for metatranscriptomic reads mapped to the selected genes for the indicated population genomes. Sample redox status for each time point encoded on left axis: sample: oxic (>90 µmol O_2; yellow), dysoxic (20-90 µmol O_2; teal), suboxic (1-20 µmol O_2; blue), anoxic (<2 µmol O_2), sulfidic (purple).

Supplementary References

1 Sanford, R. A. *et al.* Unexpected nondenitrifier nitrous oxide reductase gene diversity and abundance in soils. *Proc Natl Acad Sci U S A* **109**, 19709-19714
