# Supplementary material for: Diverse Marinimicrobia bacteria may mediate coupled biogeochemical cycles along eco-thermodynamic gradients
Source: Nat Commun. 2017 Nov 15;8:1507. doi: 10.1038/s41467-017-01376-9 (PMC5688066; doi:10.1038/s41467-017-01376-9)
Supplement: Supplementary file 3 — Description of Additional Supplementary Files [file 41467_2017_1376_MOESM3_ESM.pdf]

Description of Additional Supplementary Files

File Name: Supplementary Data 1
Description: Genomic Features of Marinimicrobia SAGs including assembly and annotation information.

File Name: Supplementary Data 2
Description: Metagenome inventory for global fragment recruitment analysis.

File Name: Supplementary Data 3
Description: Summary of recruited sequences to metagenome groups in global fragment recruitment analysis.

File Name: Supplementary Data 4
Description: Summary of relative abundance of Marinimicrobia clades recruited from metagenomes.

File Name: Supplementary Data 5
Description: Genomic features of Marinimicrobia population genome bins.

File Name: Supplementary Data 6
Description: Summary of selected metabolic functions in Marinimicrobia clades.
